# Novel approach of combined endoluminal and external vacuum therapy for wound healing disorders after salvage pharyngeal reconstruction

**DOI:** 10.1007/s00405-025-09227-w

**Published:** 2025-02-04

**Authors:** Maximilian Gaenzle, Juergen Feisthammel, Markus Pirlich, Andreas Dietz, Matthaeus Stoehr

**Affiliations:** 1 Department of Otorhinolaryngology, Head and Neck Surgery, University Hospital of Leipzig, Liebigstrasse 12, Haus 1, 04103 Leipzig, Germany; 2 Department of Oncology, Gastroenterology, Hepatology and Pneumology, University Hospital of Leipzig, Leipzig, Germany

**Keywords:** Pharyngocutaneous fistula, Salvage laryngopharyngectomy, Vacuum therapy, Endoscopic negative pressure therapy

## Abstract

**Objective:**

Patients with recurrent head and neck carcinoma are considered for salvage surgery if resectability with clear margins is achievable. However, postoperative complications such as wound healing disorders and pharyngocutaneous fistulas remain significant challenges. While various reconstructive surgical techniques exist, supportive vacuum therapy—both external and endoluminal—has been explored as an alternative treatment modality.

**Type of Study:**

We present a case report of a 60-year-old male patient with recurrent laryngeal cancer who underwent salvage laryngopharyngectomy following multiple previous surgical procedures and definitive radiochemotherapy. Due to persistent pharyngocutaneous fistula formation despite multiple reconstructions using pectoralis major flaps, an anterolateral thigh flap, and a fasciocutaneous deltopectoral flap, a novel approach combining endopharyngeal and external vacuum therapy was implemented.

**Results:**

Over six weeks, with vacuum system changes performed twice weekly, the pharyngocutaneous fistula successfully closed, leading to complete wound healing.

**Conclusion:**

This case demonstrates the potential effectiveness of a combined endopharyngeal and external vacuum therapy approach for treating persistent pharyngocutaneous fistulas when conventional surgical options are exhausted. Notably, the patient was able to resume oral food consumption following therapy, highlighting the functional benefits of this novel treatment strategy.

## Introduction

Salvage surgery in pre-irradiated head and neck areas carries higher risks of complications, including wound healing disorders, infections, and persistent fistula formation, compared to primary surgery. Reconstruction with well-vascularized, non-pretreated tissue using pedicled or microvascular flaps and effective wound management is recommended [1, 2]. Salvage laryngopharyngectomy is challenging due to tissue fibrosis, scarring, and reduced blood supply caused by radiotherapy [3]. There is no consensus on the optimal tissue transfer method for recurrent laryngeal or hypopharyngeal carcinoma. Some studies favor the pectoralis major flap, while others report better outcomes with free tissue transfer [4, 5]. Tissue from non-irradiated areas reduces fistula risk [6, 7]. However, less than half of resectable tumors remain operable after radiochemotherapy[8]. Persistent pharyngocutaneous fistulas are managed through conservative wound care, endoscopic interventions (e.g., stents or vacuum therapy), or surgical revision [9]. However, each revision increases the length of hospitalization and treatment costs and carries a new surgical risk [10]. Stents are effective mainly for acute, non-infected defects [11]. Whereas in the past, reoperation or stent implantation was usually considered in cases of insufficiency, vacuum sponge therapy has been increasingly used lately [12].

The first intracorporal application of vacuum sponge therapy was originally introduced in lower gastrointestinal fistulas in 2001, known today as Endo-Sponge-therapy (Endo-Sponge®, B.Braun, Melsungen, Germany) [12]. It enhances healing by improving perfusion, inducing granulation, reducing bacterial colonization, and approximating wound edges [13, 14]. To date, there is no standardized application of negative pressure therapy in the head and neck region published. Merely case reports and case series document its use primarily for orocutaneous or pharyngocutaneous fistulas and saliva contamination. However, the therapy is applied externally as exo-sponge, known as negative pressure wound therapy (NPWT), with a high healing success in terms of fistula closure of 78 to 100% [15]. A few groups describe the use of endoscopic negative pressure therapy (ENPT) in the head and neck region with negative pressure of 125 mmHg, mainly for primary salivary fistulas after laryngectomies, partly combined with flap plasty from the outside of the defect [16, 17].

In this report, we describe first time in literature the novel approach of the simultaneous application of an endoluminal and external vacuum system for large fistula closure in the head and neck region, which turned out to be successful.

## Case report

We were confronted with the case of a 60-year-old male patient who was initially pretreated alio loco for glottic laryngeal carcinoma. The initial diagnosis of a glottic laryngeal carcinoma of the left vocal cord was staged pT2 pN0 cM0 and treated by radiofrequency ablation chordectomy 3 years before presenting in our hospital. Due to a R1 situation, an open partial frontolateral laryngeal resection (according to Le Roux-Robert) and simultaneous bilateral selective neck dissection was performed shortly thereafter. Due to respiratory insufficiency and prolonged ventilation, a tracheostomy had to be performed. One year later, a recurrent laryngeal carcinoma of the epiglottis was detected (rcT3 cN0 cM0), treatable with laryngectomy but according to the patient’s decision treated with definitive combined bilateral radiochemotherapy of the cervical lymphatic drainage areas with 60.0 Gy, a sequential boost of the tumor to 70.0 Gy and a simultaneous chemotherapy with 3 cycles cisplatin 10 mg/m^2^ BSA. Another 11 months later, the patient presented to our hospital with a supraglottic recurrence infiltrating the residual epiglottis, the prelaryngeal skin with tumor fistula above the preexisting tracheostomy. It was staged yrcT4a yrcN2b cM0 and discussed in the interdisciplinary head and neck tumor board and recommended for salvage laryngopharyngectomy, selective neck dissection (SND) on the left and modified radical neck dissection (mRND) on the right side. The operation was performed with defect coverage by a pectoralis major pedicled flap from the left thorax. To maintain the openness of the neopharynx, we inserted a salivary bypass tube (Montgomery®, Bess Medizintechnik Gmbh, Berlin). Altogether, after histopathological processing, an R0 situation was achieved (pT4a pN0 (0/1) L0 V0 Pn0, UICC IVA, R0, squamous cell carcinoma). Adjuvant radiotherapy was recommended by the postoperative tumor board, but could not be performed due to postoperative wound healing complications described below.

**Fig. 1:**
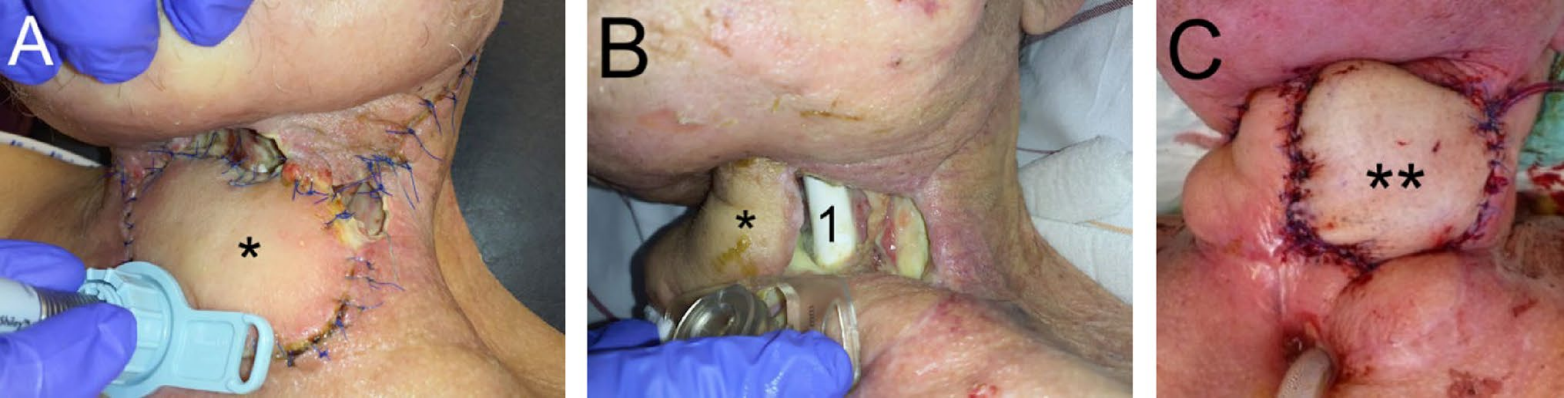
**A** Development of two fistula sites cranial and lateral of the pectoralis flap from the left (*) 2 weeks after salvage-laryngectomy, **B** after another 2 weeks under conservative wound management now measuring 8 × 8 cm with open view to the inserted salivary bypass tube (1) and **C** after insertion of the anterolateral thigh (ALT) flap (**)

During treatment, the patient developed two pharyngocutaneous fistulas: cervically on the left side and cranially of the pectoral flap, both initially measuring approximately 2 × 2 cm in size, see Fig. 1A. The defect sites were treated with sterile wound dressings, calcium alginate fleece (Trionic®, 4 M Medical GmbH, Norderstedt) and a hydropolymer dressing (Polymem Wic®, mediset clinical products GmbH, Mettmann) with daily changes. Despite inhibition of salivation with scopolamine patches (Scopoderm TTS®, Baxter Deutschland GmbH Medication Delivery, Unterschleißheim) and 50 IE botulinum toxin injection (Xeomin®, Fa. Merz Therapeutics GmbH, Frankfurt am Main) into both parotid glands, surgical wound revisions did not prevent progressive fistula development up to a size of 8 × 8 cm (Fig. 1B). Four weeks after the laryngopharyngectomy, the defect was plastically covered using a pedicled pectoralis major flap from the right side. Shortly after, wound healing disorders with a renewed large fistula developed again. After repeated revisions and later wound consolidation with antibacterial dressing, the defect was again closed with a microvascular anastomosed anterolateral thigh (ALT) flap from the right another 2 month after the closure with the pectoralis flap from the right (Fig. 1C).

**Fig. 2:**
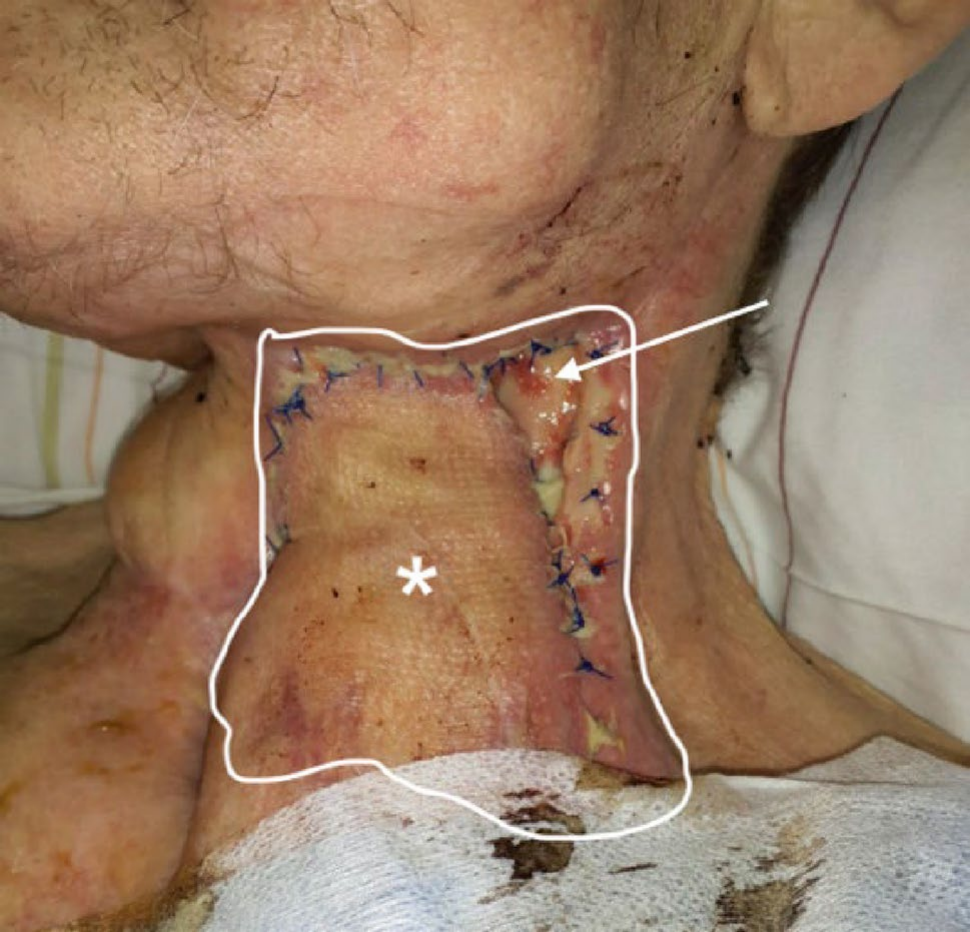
Defect after the removal of the ALT flap (white outlined area), newly inserted deltopectoral flap (*), incipient wound dehiscence after left deltopectoral flap (arrow), initially measuring 2 × 3 cm but extending to the entire craniolateral edge of the flap

However, this also resulted in wound dehiscence and partial graft loss and finally due to superinfection of the flap with high infection parameters resulting in the removal of the ALT flap. After another 3 months, plastic coverage was achieved with a pedicled fasciocutaneous deltopectoral flap from the left as a transposition flap, which, however, showed wound dehiscence and profound fistula again, being progressive despite another surgical revision, so that renewed graft loss was imminent. The defect was located in the neopharynx and extended over an area of 16 cm lateral and 10 cm medial the flap, as the entire musculocutaneous flap was dehiscent (Fig. 2).

The initial salvage-laryngopharyngectomy was conducted in early March 2023 and the last surgical wound revision of the deltopectoral flap dated to the end of September 2023. So, over a time period of 7 months of ongoing conservative and surgical wound therapy with intermediate pauses for recovery a sufficient wound closure was not achieved and the situation seemed to be hopeless. Already during that time, the patient was nourished via a percutaneous endoscopic gastrostomy tube to ensure adequate nutritional intake during the treatment period.

**Fig. 3:**
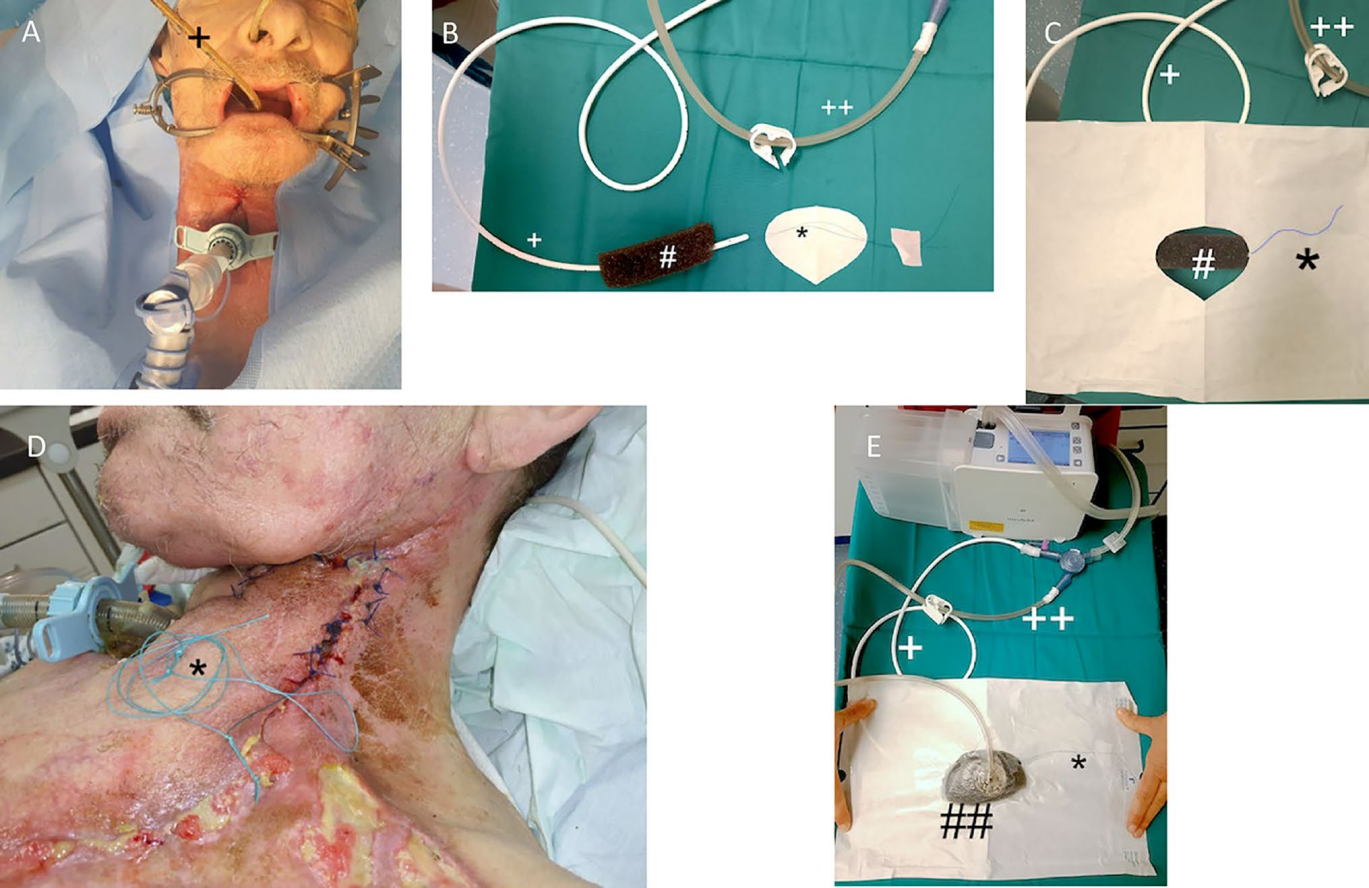
**A**–E Illustration of the endoluminal and exoluminal NPWT procedure.. Step 1 (**A**): The nasogastric tube ( +) was inserted transnasally into the patient and externalized orally. Step 2 (**B**): The endo-sponge (#) was attached to the nasogastric tube ( +) using surgical sutures. A holding suture (* (for better visibility on white background)) was attached to its end. Step 3 (**C**, **D**): The system was now reinserted transorally into the patient and the end of the nasogastric tube was positioned through the open neck wound using a Maier Sponge Forceps (shown schematically in C) at the site of the open wound and fine-tuned using the holding suture (*). The wound was then closed with surgical sutures (**D**). Step 4 (E): The wound was now covered externally with the exo-sponge (##). Both the nasogastric tube and the suction tube of the exo-sponge were connected to the suction pump using a Y-connector. Step 5: Further sponge changes were facilitated without reopening the wound. The exo-sponge was removed. Then the nasogastric tube was slightly retracted, and the endo-sponge was externalized transorally. The holding suture remained securely in place at the tip of the nasogastric tube. The old endo-sponge was then replaced by a new one and reattached to the tube again. Using the holding suture, the entire system was carefully repositioned through the already sutured neck wound. At no point was the use of forceps or re-opening of the wound necessary. Finally, the exo-sponge was attached again

In an interdisciplinary approach with colleagues from the department of gastroenterology, we planned a combined vacuum treatment. Within one surgical procedure, we first refreshed the wound edges but did not readapt them yet. Afterwards, a gastric tube Charrier 16 was inserted transnasally and passed out orally (see Fig. 3A). Then, the pharyngeal end was connected to the vacuum sponge, which was adapted to the shape of the neopharynx by the surgeon and then retracted into the neopharynx. To retract the sponge into the neopharynx, we tied a holding suture (Polyethylen 2–0) to the pharyngeal end of the tube (where the sponge was already attached) and manually pulled it from the outside through the persisting fistula before ultimately closing the wound with sutures (see Fig. 3D). The holding suture facilitated subsequent endo-sponge changes in the following operations. By adequately filling the neopharynx lumen from the inside using the endo-sponge and applying an additional sponge externally (in conjunction with adhesive film covering), a sufficient negative pressure was created. This approach allowed for improved tissue healing through granulation tissue formation. Further aboral, the salivary bypass tube maintained the openness of the neopharynx.

**Fig. 4:**
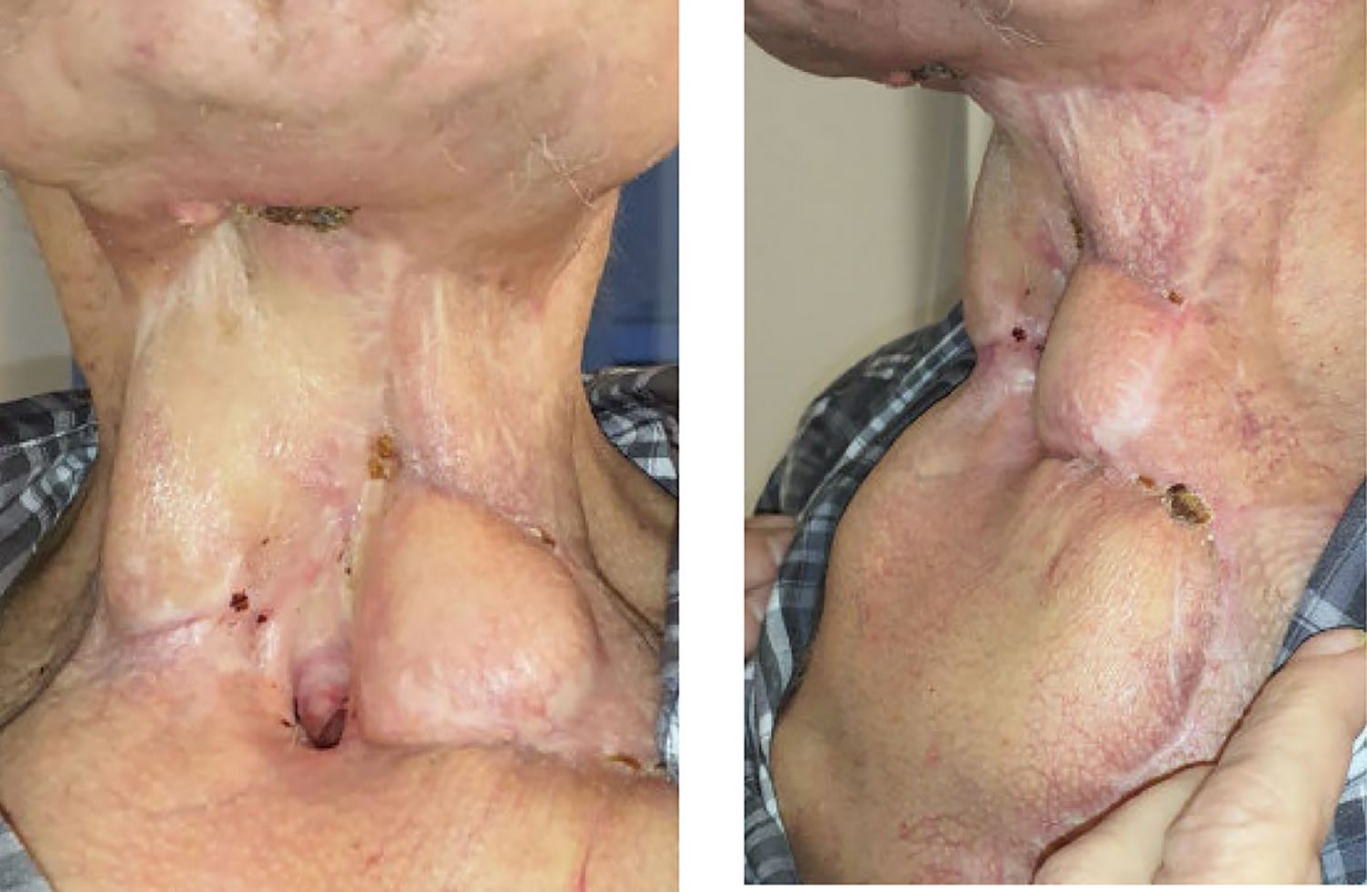
Completion of vacuum suction therapy and first follow up 4 weeks later. The initial salvage laryngopharyngectomy was performed in March 2023. Vacuum therapy was applied from October to November 2023. In January 2024, the pedicle of the deltopectoral flap was transected, and the salivary bypass tube was removed simultaneously

Due to the anatomical situation and minimal leaks, this was nevertheless subject to air leakage. The regular KCI-suction pump® (3 M Deutschland GmbH, Neuss) therefore displayed persistent error messages with automatic shutdowns and had to be replaced by a continuous Medela-suction-system® (Type Invia Liberty, Medela Medizintechnik GmbH & Co. Handels KG, Eching). This system tolerated a higher air leakage at a suction of – 75 mmHg without interrupting the suction performance. Higher pressures were not tolerated by the system. The two sponges were connected via a Y-connection so that only one pump was required. We changed the vacuum sponges regularly, initially twice a week under general anesthesia, later only once a week, as we noticed a positive wound healing. In total, thirteen vacuum dressing changes were conducted under general anesthesia until the fistula was successfully closed after 6 weeks of therapy (see Fig. 4). Tissue samples of several sites around the fistula were taken once during the procedure showing no signs of malignancy. Systemic antibiotics only had to be administered at the beginning of the treatment according to inflammation parameters.

After the removal of the salivary bypass tube and the pedicle transection of the deltopectoral flap in January 2024, the patient exhibited no new fistula symptoms. In regular three-monthly follow-up visits after the treatment concluded, the patient was able to swallow liquids and small solid pieces. Ongoing dressing changes became unnecessary, facilitating the patient's transfer from a nursing home to his own residence.

## Discussion

For the closure of pharyngocutaneous fistulas, the literature describes various therapy options, including conservative wound management, surgical flap techniques, endoluminal stenting, and vacuum therapy [1, 5, 17, 18]. Surgical closure often relies on microvascular flap surgery. Patel et al. (2013) reported lower fistula rates with pectoralis major flap reconstruction (15%) compared to free flap reconstruction (25%) or primary closure (34%). Fistula persistence was also shorter with pectoralis major flaps (9 weeks) and free flaps (6.5 weeks) compared to primary closure (14 weeks) [4]. Piazza et al. (2021) documented low fistula rates (5.4%) using radial forearm and anterolateral thigh (ALT) flaps [5]. Despite sequential use of pectoralis major, ALT, and deltopectoral flaps, poor wound healing in our case necessitated alternative approaches.

Endoluminal stent therapy presented here the advantage of maintaining continuous oral food intake through the intact pharyngeal lumen. But it is less effective in infected areas and poorly tolerated in complex upper gastrointestinal defects [19, 20]. Furthermore, stents can worsen fistula size in heavily pretreated regions [20]. Therefore, stent therapy was deemed unsuitable for our patient.

NPWT, applied endoscopically as ENPT, has shown superior success rates over reoperation or stenting in gastrointestinal anastomotic leakage, with lower complication rates and higher healing success [21]. Studies report optimal sponge changes every 3–5 days over a mean of 22 days, achieving a 94% healing rate [22]. In the head and neck region, ENPT has been used for salivary fistulas with pressures around -125 mmHg [16, 17]

In our case, relying solely on isolated endoluminal treatment seemed to be ineffective. The fistula exposed the sponge to air so that a sufficient vacuum could not have been created. Multiple prior surgeries, along with persistent saliva, prevented the closure of the external wound using only standard adhesive film. Furthermore, ongoing contact with saliva contributed to recurring healing issues. Isolated external vacuum also seemed not to be promising as the connection of the cervical wound to the pharynx had led to the same air leakage. As practiced by Asher et al.[17], a flap from the outside as counterpart to the ENPT was after already witnessed losses of two pectoralis flaps, one ALT flap and a renewed imminent graft loss of the deltopectoral flap not feasible. Our situation necessitated the sealing and elimination of saliva along all wound edges, both endoluminally and externally, while maintaining an open neopharynx and creating a tight vacuum.

Therefore, a combined approach was adopted, sealing the wound both endoluminally and externally while maintaining an open neopharyngeal lumen via a salivary bypass tube. Saliva production was reduced using intraparotideal botulinum toxin (50 IU) and scopolamine patches [23, 24], though scopolamine dosage had to be adjusted due to mild agitation. Despite initially aiming for the described negative pressure of 125 mmHg [2, 22], practical considerations led us to adapt to – 75 mmHg due to the suction system's intolerance to higher pressures caused by air leakage. Sponge changes, typically conducted under local anesthesia as described in literature [25], were found to be too painful. Specifically, endoluminal changes induced a gag reflex affecting the newly formed tissue, necessitating all changes to be performed under general anesthesia, which the patient tolerated well.

Over 42 days and 13 sponge changes, the therapy successfully closed the fistula. Cancer recurrence was ruled out through biopsies. Nutritional support was maintained via a percutaneous endoscopic gastrostomy tube, and oral food intake was restored 2 months post-therapy. Compared to reported mean treatment durations of 3.7–23 days in cases with smaller fistula [15, 17, 26], our case required a longer period due to the large fistula size (16 × 10 cm). Sponge changes were adjusted based on vacuum pump performance and air leakage. Photographic documentation, close patient communication, and high compliance were critical for success. Managing the persistent suction pump noise could require low-dose sedative psychotropic drugs and noise-canceling headphones.

Future considerations may explore outpatient approaches. However, challenges include the constant presence of the nasal gastric tube, posing risks for dislocation, and the difficulty of regular nursing staff monitoring. Outpatient presentations, particularly over long distances, can be burdensome. In our case, outpatient care was unfeasible due to the patient's frailty and tranquilizer requirements.

## Conclusion

The combined use of external and (tube-guided) endopharyngeal vacuum therapy significantly improves wound healing, especially for large head and neck fistulas. While time-intensive, this approach enhances the patient's quality of life by restoring oral food intake and reducing the need for repeated surgical interventions. This novel treatment strategy should be considered a valuable addition to the therapeutic options for salivary fistulas in the head and neck region.

## Data Availability

The data that support the findings of this study are available from the corresponding author, Maximilian Gaenzle, upon reasonable request.
